# Dose-Dependent Dual Role of PIT-1 (POU1F1) in Somatolactotroph Cell Proliferation and Apoptosis

**DOI:** 10.1371/journal.pone.0120010

**Published:** 2015-03-30

**Authors:** Nicolas Jullien, Catherine Roche, Thierry Brue, Dominique Figarella-Branger, Thomas Graillon, Anne Barlier, Jean-Paul Herman

**Affiliations:** 1 Aix-Marseille Université, CNRS, UMR7286, 13015 Marseille, France; 2 Laboratory of Molecular Biology, APHM Conception, 13385 Marseille, France; 3 Department of Endocrinology, APHM Timone, 13385 Marseille, France; 4 Laboratory of Neuropathology, APHM Timone, 13385 Marseille, France; 5 Department of Neurosurgery, APHM Timone, 13385 Marseille, France; University of Cordoba, SPAIN

## Abstract

To test the role of wtPIT-1 (PITWT) or PIT-1 (R271W) (PIT271) in somatolactotroph cells, we established, using inducible lentiviral vectors, sublines of GH4C1 somatotroph cells that allow the blockade of the expression of endogenous PIT-1 and/or the expression of PITWT or PIT271, a dominant negative mutant of PIT-1 responsible for Combined Pituitary Hormone Deficiency in patients. Blocking expression of endogenous PIT-1 induced a marked decrease of cell proliferation. Overexpressing PITWT twofold led also to a dose-dependent decrease of cell proliferation that was accompanied by cell death. Expression of PIT271 induced a strong dose-dependent decrease of cell proliferation accompanied by a very pronounced cell death. These actions of PIT271 are independent of its interaction/competition with endogenous PIT-1, as they were unchanged when expression of endogenous PIT-1 was blocked. All these actions are specific for somatolactotroph cells, and could not be observed in heterologous cells. Cell death induced by PITWT or by PIT271 was accompanied by DNA fragmentation, but was not inhibited by inhibitors of caspases, autophagy or necrosis, suggesting that this cell death is a caspase-independent apoptosis. Altogether, our results indicate that under normal conditions PIT-1 is important for the maintenance of cell proliferation, while when expressed at supra-normal levels it induces cell death. Through this dual action, PIT-1 may play a role in the expansion/regression cycles of pituitary lactotroph population during and after lactation. Our results also demonstrate that the so-called “dominant-negative” action of PIT271 is independent of its competition with PIT-1 or a blockade of the actions of the latter, and are actions specific to this mutant variant of PIT-1.

## Introduction

POU1F1 (PIT-1), is a pituitary- and lineage-specific POU homeodomain transcription factor that was among the first vertebrate transcription factors identified as having a determinant role in the development of specific cell lineages [1,2]. It has a crucial role in the terminal differentiation and expansion of the somatolactotroph and thyrotroph lineages during pituitary development as well as in the physiological regulation of the expression of the genes of prolactin (PRL), growth hormone (GH) and thyroid-stimulating hormone (TSHß) [3]. In accordance with this role, mutations of *Pit-1* lead to Combined Pituitary Hormone Deficiency (CPHD), characterized by the lack of production of PRL, GH and TSHß due to the absence of the corresponding pituitary lineages [4–6].

A number of evidences suggest that PIT-1 plays a role in the regulation of proliferation and/or survival of its target cell populations. One is the fact that mutations of *Pit-1* lead to a marked pituitary hypoplasia, with loss of the somatolactotroph and thyrotroph lineages [7], related to a decrease in cell proliferation and increase in cell death [8]. A more direct evidence reported soon after the discovery of *Pit-1* is that an asRNA specific for *Pit-1* leads to a decrease of cell proliferation in a rat somatolactotroph cell line [9]. More recently we have shown that the expression of dominant negative pathogenic form of PIT-1, PIT-1(R271W) (PIT271) leads to cell death, supporting the hypothesis that PIT-1 is required for survival and proliferation of somatolactotroph cells [10,11]. However, seemingly contradictory results have been published more recently by the group of C. V. Alvarez [12,13] that has described that an increase of intracellular PIT-1, induced by the Ret tyrosine-kinase receptor or by direct transfection, leads to death of the cells. Moreover, recently we obtained results ourselves suggesting that PIT-1 overexpression in specific conditions could decrease cell proliferation [10].

To clarify this contradiction, we have decided to re-evaluate the role of PIT-1 and the effect of PIT271 using a new approach. For that we developed an experimental model that allowed to test the action of PIT-1 and its blockade independent of PIT271 and with more reliable tools than the asRNA's used in the original 1991 study [9], and also to isolate the action of PIT271 from its interaction with endogenous PIT-1. Note that besides allowing to address the role of PIT-1, this approach allowed also to examine whether the action of PIT271 is indeed linked to its antagonization of endogenous PIT-1 as previously hypothesized.

Our results demonstrate that, despite the appearances, there is no real contradiction between the two sets of results, as we observed that both the blockade of the expression of PIT-1 and its overexpression lead to cell death and altered proliferation. Thus, PIT-1 may switch in somatolactotroph cells, in a dose-dependent fashion, between a role of cell maintenance and one of induction of cell death. Second, we show that PIT271 induces cell death and decreases cell proliferation, confirming our earlier results. However, these actions of PIT271 are independent of its interaction with endogenous PIT-1.

## Methods

### Vector constructions

**Fig 1:**
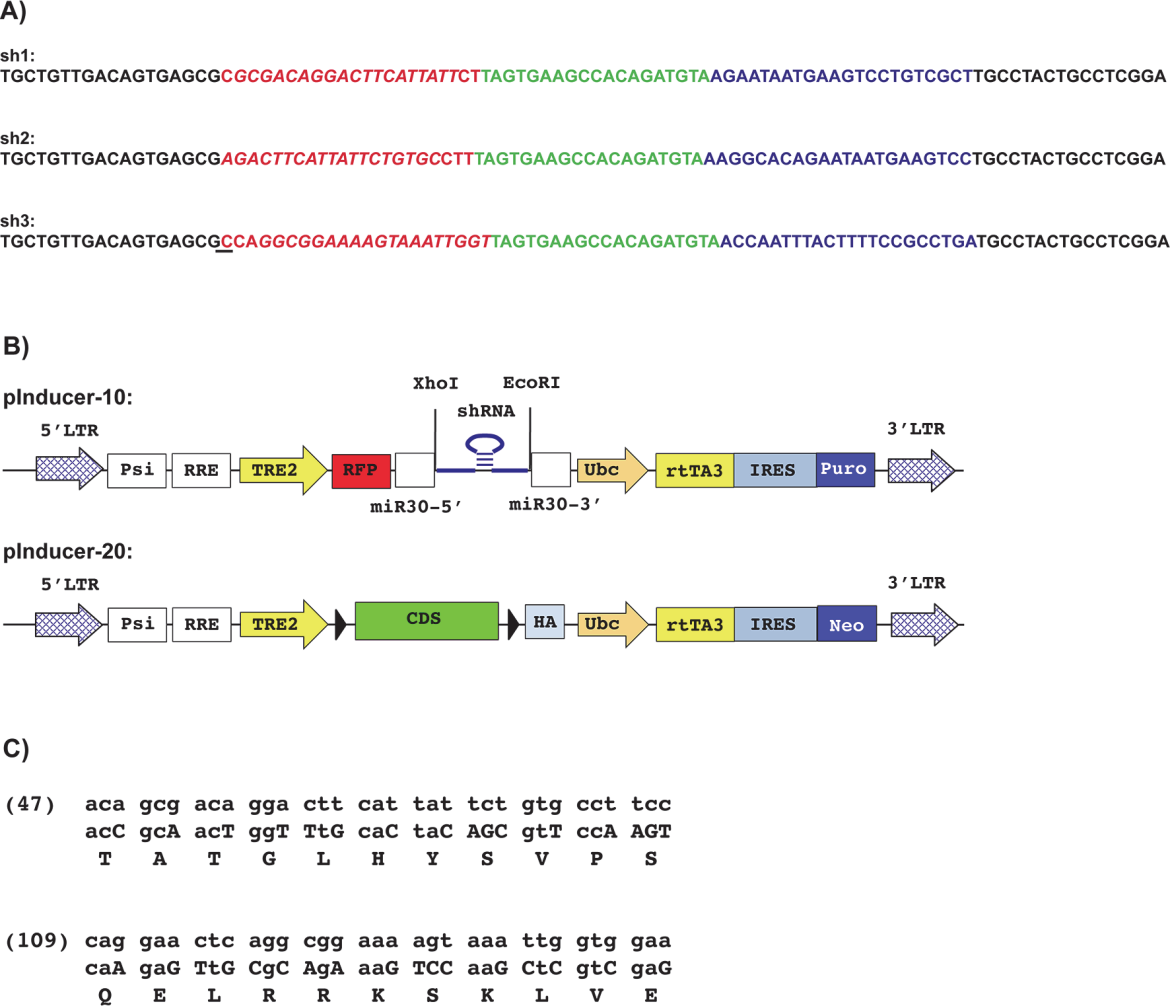
Constructs used in this study. A. Oligonucleotides used for the construction of the shRNA expressing lentiviral vectors. In red the target sequence, in blue the antisense (guide) strand, in green the sequence corresponding to the miR-30 'head'. The sequence of the Ambion siRNA on which a given shRNA construct was based is indicated in italics, underlined is the base that has been modified in order to introduce a bulge into the miRNA produced. B. Scheme of the pInducer lentiviral vectors used [15]. Expression of the red fluorescent protein (RFP) and the coupled miR30 construct containing the inserted shRNA sequence (pInducer-10) or of the inserted CDS (pInducer-20) is driven by the synthetic TRE2 promoter regulated in a doxycycline-dependent manner by the rtTA3 transcription factor expressed constitutively by the Ubc promoter. The vectors express resistance markers, allowing selection of infected cells by puromycin or G418. C. Modifications introduced in the sequences targeted by the shRNA's in the coding sequence of PIT-1. The CDS with these modifications have been used to construct the pInducer-20 based expression vectors. The wt sequence is given in the upper lines, the modified sequence with the introduced changes indicated in capital letter in the middle lines. The lower line indicated the amino acids corresponding to the codons. The numbers in parentheses indicate the ordinal number of the first codon.

siRNA's for rat Pit-1 were ordered from Ambion. To construct the shRNA lentiviral vectors, the sequences coding for the selected siRNA's were extended by three bases and inserted into an miR-30-based oligonucleotide designed as defined by Chang et al. [14] (Fig. 1A). The oligonucleotides were then ligated into the pInducer-10 lentiviral vector [15] (Fig. 1B). Rat wild type PIT-1 (PITWT) or PIT271 expressing lentiviral vectors were constructed by inserting the coding sequences for the protein into the pInducer-20 lentiviral vector, downstream of the TRE2 promoter [15] (Fig. 1B). Note that to avoid subsequent targeting of the *Pit-1* mRNA transcribed from the vector by co-expressed *Pit-1* shRNA's, conservative codon changes were introduced into the cDNA sequences corresponding to the regions targeted by the shRNA's in endogenous *Pit-1* (Fig. 1C). Lentiviral vectors were produced in 293T cells. Lentiviral vectors transducing eGFP, hPITWT or hPIT271 have been described earlier [11].

### Analysis of gene expression

For RT-qPCR cells, total RNA was isolated and purified from cells using Trizol (Life Technologies) according to the procedure given by the manufacturer. Following reverse transcription using random primers and M-MLV reverse transcriptase, qPCR was performed using an ABI 7500Fast station and the SYBR-Green PCR master mix (2x Fast Master Mix kit from KAPA Biosystems). Specific primers were designed with AmplifX (http://crn2m.univ-mrs.fr/pub/amplifx-dist). Normalization of the results for *Pit-1* was done in two steps: first using known quantities of a *Pit-1* plasmid used as external standards to obtain an evaluation of the absolute number of copies, second relative to actin to compensate for technical variations. Note that the results were the same when quantification was achieved through normalization relative to the quantity of input RNA. We used two different forward primers for *Pit-1* (or *Pit271*) targeted to the sequence that has been modified for the expression from the lentiviral vector (see above), permitting separate measurements and direct comparison of endogenous and exogenous mRNA's. Note that we have verified that the RT efficiencies were equivalent for these two mRNA species. Quantification for genes other than *Pit-1* was done using the ΔΔCt method. Expression analysis in human pituitary adenomas was performed by RT-qPCR using a TaqMan assay with specific probes, and using ß-glucuronidase as normalizing gene. All primers and probes are listed in Table 1.

**Table 1:** List of primers used for RT-qPCR quantification.

Target	Primers
*Actin*	Fw: 5'-ttgctgacaggatgcagaaggaga
	Rev: 5'-tcgtactcctgcttgctgatcca
*Aatf*	Fw: 5'-aatgctcacctgaaggacttggac
	Rev: 5'-gaaactcaggagcttgctaagcac
*Bag1*	Fw: 5'-acagtgagccaattgtccaagacc
	Rev: 5'-acagatacctccaagtccttcagc
*Bag3*	Fw: 5'-tccgaccaggttacattcccattc
	Rev: 5'-gaggacgaggatgagcagtcag
*ß-Gus*	Fw: 5'-gaaaatatgtggttggagagctcatt
	Rev: 5'-ccgagtgaagatccccttttta
	P: 5'-fam-ccagcactctcgtcggtgactgttca-tamra
*Ccni*	Fw: 5'-agacagtttctgtggatgttcctc
	Rev: 5'-agaagttggttgcaggccatacag
*Dad1*	Fw: 5'-cagcttcatcttagcggtttgc
	Rev: 5'-ctgccagctcaagaattctgatcc
*Dap*	Fw: 5'-aaagtcgctgggattcggattgtg
	Rev: 5'-gctgttggatatgctgcgttcttg
*NudC*	Fw: 5'- ttggttaccagtgaccccgaaatc
	Rev: 5'- actgcaagagttcaggaaggtg
*p19Arf*	Fw: 5'- tgcagattcgaactgcgagga
	Rev: 5'-aggcatcgcgcacatccag
*p53*	Fw: 5'-tttgtgcctgtcctgggaga
	Rev: 5'-ttccaaggcctcattcagctct
*hPit-1*	Fw: 5'- tgcaaactgaaagcaatattatcc
	Rev: 5'-ttcttcgttttcttttcctttcattt
	P: 5'-fam-atggctggaggaagctgagcaagtaggag-tamra
*rPit-1*, *endogenous*	Fw: 5'-ccacagcgacaggacttcatta
	Rev: 5'-aggggaggaaacccatgact
*rPit-1 SI resist (wtPit/ Pit271)*	Fw: 5'-cgcaactggtttgcactaca
	Rev: 5'-aggggaggaaacccatgact
*PRL*	Fw: 5'-aattagccaggcctatcctgaagc
	Rev: 5'-tggacaatttggcacctcagga
*Rad17*	Fw: 5'-aggaaggctgcctttagaacagac
	Rev: 5'-tgaatcccaagctcctttgacag
*Serinc3*	Fw: 5'-gtgacgtgctggtcggttttaaag
	Rev: 5'-attccaagagtgagccaggtctac
*Sod2*	Fw: 5'-gtgaacaatctgaacgtcaccgag
	Rev: 5'-ttgcagtgggtcctgattagagc
*Tnfrsf1a*	Fw: 5'-ggtggagatttctccttgcaaagc
	Rev: 5'-aggaagataaccagaggcaacagc
*Vcp*	Fw: 5'-tctggagccgattcaaaaggtg
	Rev: 5'-atggctggatgctgatgacatctc

### Western-blots

Cell extracts were prepared using a buffer containing Tris-HCl (50 mM pH 7,4), NaCl (150mM), EDTA (1mM), NP40 (1%), deoxycholate (0,25%) and "Complete Protease Inhibitor" cocktail (Roche, 0.4%). Proteins (10 μg per lane) were separated by SDS-PAGE and transferred onto PVDF Low Fluorescence membrane (Millipore). Proteins were detected by incubation with primary antibodies against PIT-1 (mouse monoclonal clone A-1, Santa-Cruz, 1:700), PRL (rabbit polyclonal, A.F. Parlow, National Hormone and Pituitary program, 1:200 final dilution), p19ARF (rabbit polyclonal 07–543, Upstate, or rabbit polyclonal ab80 Abcam, final dilution 1:1000 for both), P53 (monoclonal C12 Cell Signaling, or monoclonal PAb421 Calbiochem, final dilution 1:1000 for both) or ß-actin (rabbit polyclonal AC-15, Sigma, 1:5000 final dilution), followed by incubation with secondary antibodies coupled to Q-dots (Q-Dot 655 anti-rabbit or Q-Dot 565 anti-mouse IgG conjugates (Molecular Probes, 1:1000 final dilution), and visualization using GBox XT4 (Syngene) with UV epi-illumination and specific filters (665BP20 and 565BP20), or coupled to fluorescent labels (Alexa-Fluor 647 or 555, Molecular Probes, 1:1000 final dilution), and visualization using Typhoon FLA9000 (GE Healthcare). Quantification of bands was done with ImageQuant TL (GE Healtcare)

### Cell culture

Rat somatolactotroph GH4C1 cells (ATCC) were grown in F10 medium (Life Technologies) supplemented with horse serum (15%) and fetal bovine serum (5%) at 37°C in the presence of 5% CO_2_. African green monkey kidney CV1 cells (ATCC) were grown in DMEM (Life Technologies) supplemented with fetal bovine serum (10%) at 37°C in the presence of 5% CO_2_.

To evaluate the effect of siRNA's on the expression of *Pit-1*, cells on 24-well plates were transfected with 60 pmol/well siRNA (final concentration 100 nM), using Lipofectamine (Life Technologies) according to the manufacturer's instructions, and lysed 48h later.

Lentiviral infection was done using the filtered viral supernatants of the 293T producer cells, in the presence of 4 μg/ml Polybrene. Antibiotic selection (pInducer-10-based vectors: puromycin, 10 μg/ml, pInducer-20-based vectors: G418, 600 μg/ml) was started two days after infection. To obtain shRNA-expressing clones, 1000 puromycin-resistant cells were plated onto 100 mm plates and clones were picked manually. Induction of shRNA expressed by pInducer-10, or of *Pit-1* or *Pit271* expressed by pInducer-20 was obtained by adding doxycycline to the medium. The inducer was maintained for the whole duration of the experiment and refreshed every second day.

To evaluate proliferation, 30 000 cells/well (GH4C1 cells) and 15 000 cells/well (CV1 cells) were plated on 24-w plates. Doxycycline (1 μg/ml) was added next day (D0) to induce expression of shRNA and/or of *Pit-1* or *Pit271* and maintained throughout the experiment. Cells were counted starting on D0 using the Specter cell counter (Millipore) from triplicate wells. Experiments were repeated four times. Statistical analysis was performed on the data obtained for D5 in the four experiments, using the number of cells following doxycycline treatment relative to that obtained without doxycycline.

Cell division rate was examined on cultured cells using the Click-iT EDU essay (Life Technologies), while cell death was evaluated using the Apoptag Tunel kit (Millipore). When using the Click-iT EDU assay, EDU was added on day 3 of induction by doxycycline, 15–17 h before fixing the cells. Labeled and unlabeled cells were counted from 6 micrographs taken at random from duplicate wells and the experiment was repeated two (cell death assay) or three (division rate) times.

### Human pituitary adenoma culture

Human pituitary adenoma samples were obtained following surgical resection, following approval by the Ethics Committee of the University of Aix-Marseille II (France) and having obtained written informed consent from each patient to participate in this study. Ten adenomas, described in detail earlier [10], were used (A1-A6: somatotroph or somatolactotroph adenomas, P1 and P2: prolactinomas, X2 and X3: nonfunctional adenomas). Methods for culture of human pituitary adenomas have been described in detail [10]. Cells were infected after 5 days of culture with lentiviral vectors at a multiplicity of infection of 10, in the presence of 8 μg/ml Polybrene, resulting in an infection rate of 80–96% [16]. Viability assay, using Cell Titer-Glo (Promega) was conducted 9 days post-infection.

### Flow cytometry

For cell cycle analysis, cells were trypsinized, washed, fixed and permeabilized overnight using 70% ethanol at -20°C. Following centrifugation, cells were suspended in a solution containing EDTA (20mM), Tween-20 (0.5%), RNAse (100 μg/ml) and propidium iodide (10 μg/ml, Sigma). For dye-exclusion based viability assay, live cells were suspended, following trypsinization and centrifugation, in normal medium containing To-Pro3 (75 nM, Life Technologies). Analysis was performed on a BD FACS Canto analytical flow cytometer.

### Statistical analysis

Results are presented as mean ± SEM. Statistical analysis by ANOVA followed by post-hoc Tukey HSD test for multiple comparisons or by regression analysis was performed using the OpenStat free statistical package (http://statpages.info/miller/OpenStatMain.htm).

## Results

### Conditional blockade and exchange of endogenous PIT-1

Our aims was to test the role of PIT-1 on the one hand, and to evaluate, on the other hand, whether cell death observed upon expression of PIT271 [11] is related to its antagonization of endogenous PIT-1 or is a direct action, independent of its interaction with endogenous PIT-1. To achieve our aims, we used, as in the earlier studies, the GH4C1 rat somatolactotroph cell line and we blocked endogenous *Pit-1* using shRNA's on the one hand, or exchanged endogenous PIT-1 for PITWT or PIT271 on the other hand. This latter exchange approach allowed to evaluate the action of PIT271 independent of its interaction with endogenous PIT-1. However, given that these modifications could result in cell death, we used conditional systems, relying on expression vectors incorporating a modified doxycycline-regulated system (Fig. 1B.) [15,17].

**Fig 2:**
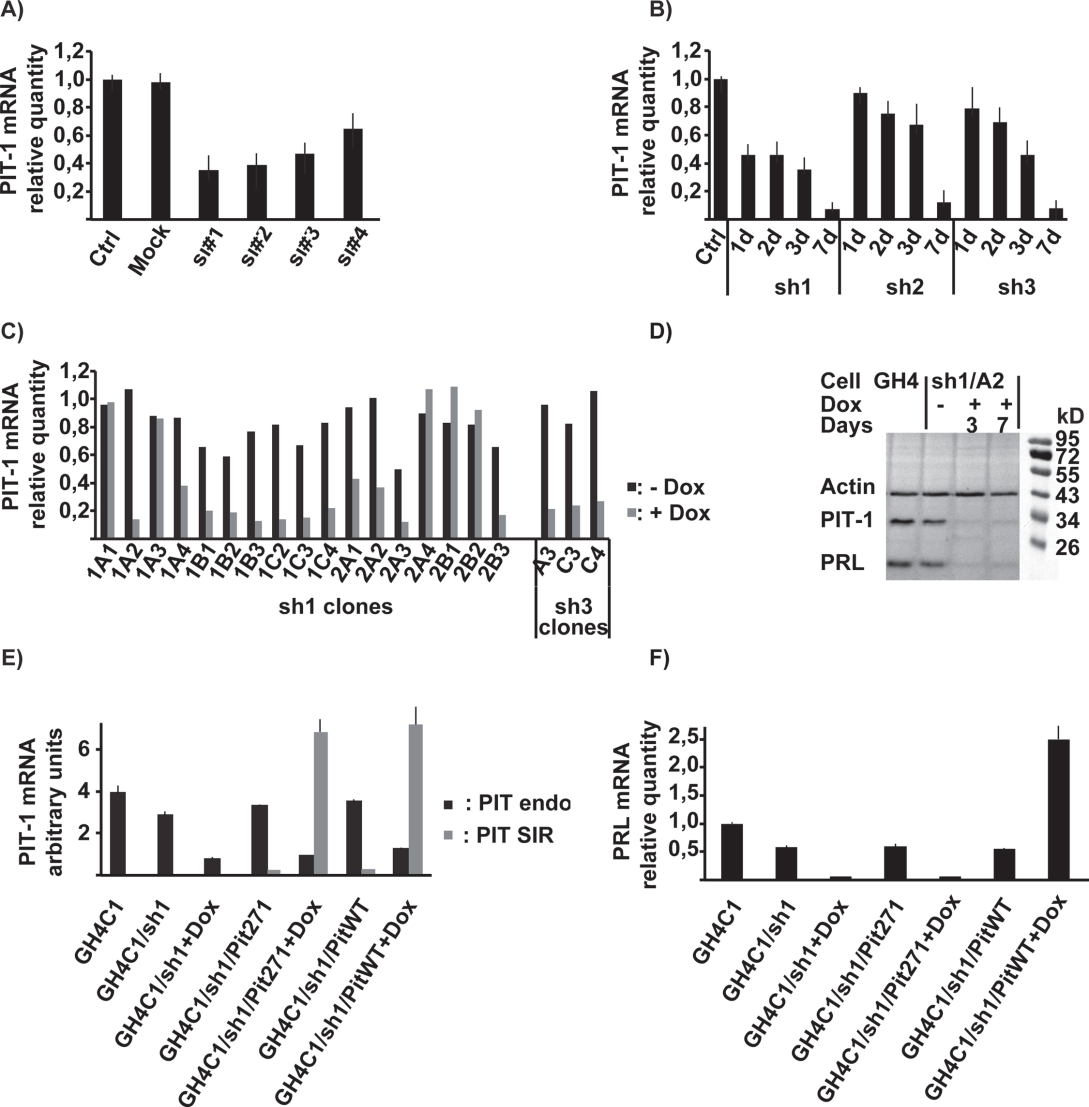
Development of GH4C1 cells clones with inducible expression of shRNA and/or PITWT and PIT271. A. Pit-1 mRNA levels in GH4C1 cells 48 hours after transfection of siRNA's (Ambion) specific for rat Pit-1. Results are expressed relative to levels found in Control ('Ctrl') cells corresponding to non-transfected cells and correspond to the mean ± SEM of two experiments. 'Mock' corresponds to GH4C1 cells transfected with an unrelated non-specific control siRNA. B. Time-course of PIT-1 mRNA levels following induction of shRNA expression in GH4C1 cells (polyclonal population) infected with shRNA expressing conditional lentiviral vectors. Results are the mean ± SEM of two experiments and are expressed relative to levels found in Control ('Ctrl') cells corresponding to GH4C1 cells infected with the sh1 expressing vector and not exposed to the inducer doxycycline. Induction was achieved by exposure to 1μg/ml doxycycline for 1 to 7 days. C. PIT-1 mRNA levels in individual clones of GH4C1 cells harboring the shRNA expressing conditional lentiviral vectors, 6 days after induction of shRNA expression by 1 μg/ml doxycycline. Results are expressed relative to values found for GH4C1 control cells. D. Western blots of control GH4C1 cells and GH4C1/sh1 A2 clone. Induction by 1 μg/ml doxycycline was maintained for 3 or 7 days. Molecular weight ladder (Thermo Page Ruler) on the right. E. Expression of mRNA's for PIT-1 or PIT271 in the different cell lines in the absence or presence of 1 μg/ml doxycycline for 6 days. 'Pit endo' corresponds to wt endogenous PIT-1, ‘Pit SIR’ (si-resistant) to PITWT or PIT271 expressed from the conditional expression vectors. The results for 'Pit endo' and ‘Pit SIR’ have been obtained by normalizing the values obtained in qPCR to those obtained with known quantities of external controls, and are thus comparable. Results are mean ± SEM of three measures. F. Expression, in the same cells as in Fig. 2E, of PRL mRNA's. Results are expressed relative to values found for GH4C1 control cells.

Conditional blockade of endogenous *Pit-1* was obtained by expressing an shRNA specific for r*Pit-1*. First, the capacity of a panel of 4 siRNA commercialized by Ambion to block expression of *Pit-1* was examined through transient transfection by measuring levels of mRNA's by RT-qPCR (Fig. 2A). Based on this test, the three siRNA's that resulted in at least 50% inhibition of *Pit-1* mRNA expression were used to define shRNA sequences and build pInducer-10 based lentiviral vectors allowing the conditional expression of shRNA. Following infection and selection of GH4C1 cells by puromycin, the efficacies of the three lentiviral constructs in term of blockade of *Pit-1* expression upon induction of shRNA were compared (Fig. 2B). At this step, sh2 turned out to have a somewhat lesser performance than the other two constructs and was abandoned. After cloning of the GH4C1-sh1 and GH4C1-sh3 populations, the individual clones were compared (Fig. 2C). Clones displayed marked differences in term of leakage of the sh vector (inhibition of endogenous *Pit-1* expression in the absence of the inducer) as well as of activity following induction, and based on this comparison the clone GH4C1-sh1/1A2 (designated in the following as GH4C1-sh1) was finally chosen for further work. Complementing the RT-qPCR results, we observed that the levels of PIT-1 and PRL proteins were indeed very significantly decreased following the initiation of doxycycline treatment (Fig. 2D). Note that measuring *Prl* expression in this and the subsequent experiments allowed us to evaluate the functional effects of our experimental manipulations on the classical downstream targets of PIT-1 of which *Prl* was taken as an example.

To exchange endogenous PIT-1 for exogenous PITWT or PIT271, the GH4C1-sh1 clone obtained above was infected with a second pInducer-20 based lentiviral construct, similar to the one used for the expression of the shRNA, but expressing neomycin resistance as selection marker. This construct expressed conditionally, still in a doxycycline-dependent manner, either PITWT or PIT271. Thus, exposure of the infected cells to doxycycline leads, in a single step, to the blockade of the expression of endogenous PIT-1 and the expression of exogenous PITWT or PIT271. Note that, to avoid interference of the shRNA with the expression of the exogenous construct, the sequences recognized by the shRNA's were modified in the latter, resulting in siRNA-resistant (SIR) *Pit-1* or *Pit271* constructs (Fig. 1C). This modification allowed also, using specific primers (Table 1), to measure subsequently separately and compare the levels of endogenous and exogenous (SIR) mRNA's.

The efficacy of this exchange is demonstrated in Fig. 2E (compare GH4C1/sh1/Pit271 vs. GH4C1/sh1/Pit271+dox and GH4C1/sh1/PitWT vs. GH4C1/sh1/PitWT+Dox). The level of expression of exogenous PITWT or PIT271 reached upon induction of the lentiviral vector is about twice the expression of endogenous PIT-1 in control cells with no shRNA expression. Note that the subsequent evaluation of protein levels by Western blots (see below) corroborated these results.

As shown previously, PRL expression fell by more than 90% in the cells (Fig. 2F) following the induction of shRNA, despite the persistence of some endogenous PIT-1 expression (around 20% of normal levels, see Fig. 2E), indicating that this residual level of PIT-1 expression is not sufficient to achieve a significant functional effect. The replacement of endogenous PIT-1 by exogenous PITWT reinstated PRL expression (Fig. 2F), that reached a level of expression higher than that observed in control cells, in accordance with the fact that the level of PITWT expressed by the lentiviral vector following induction by doxycycline was also higher than the level of endogenous PIT-1 in control cells. On the contrary, the replacement of PIT-1 by PIT271 did not reverse the disappearance of PRL expression induced by the concurrent expression of shRNA (Fig. 2F).

For control purposes GH4C1 and CV1 cells expressing the same conditional PITWT and PIT271 vectors have also been established.

### Blockade of PIT-1 expression, but also over-expression of PITWT and PIT271 interferes with cell growth

**Fig 3:**
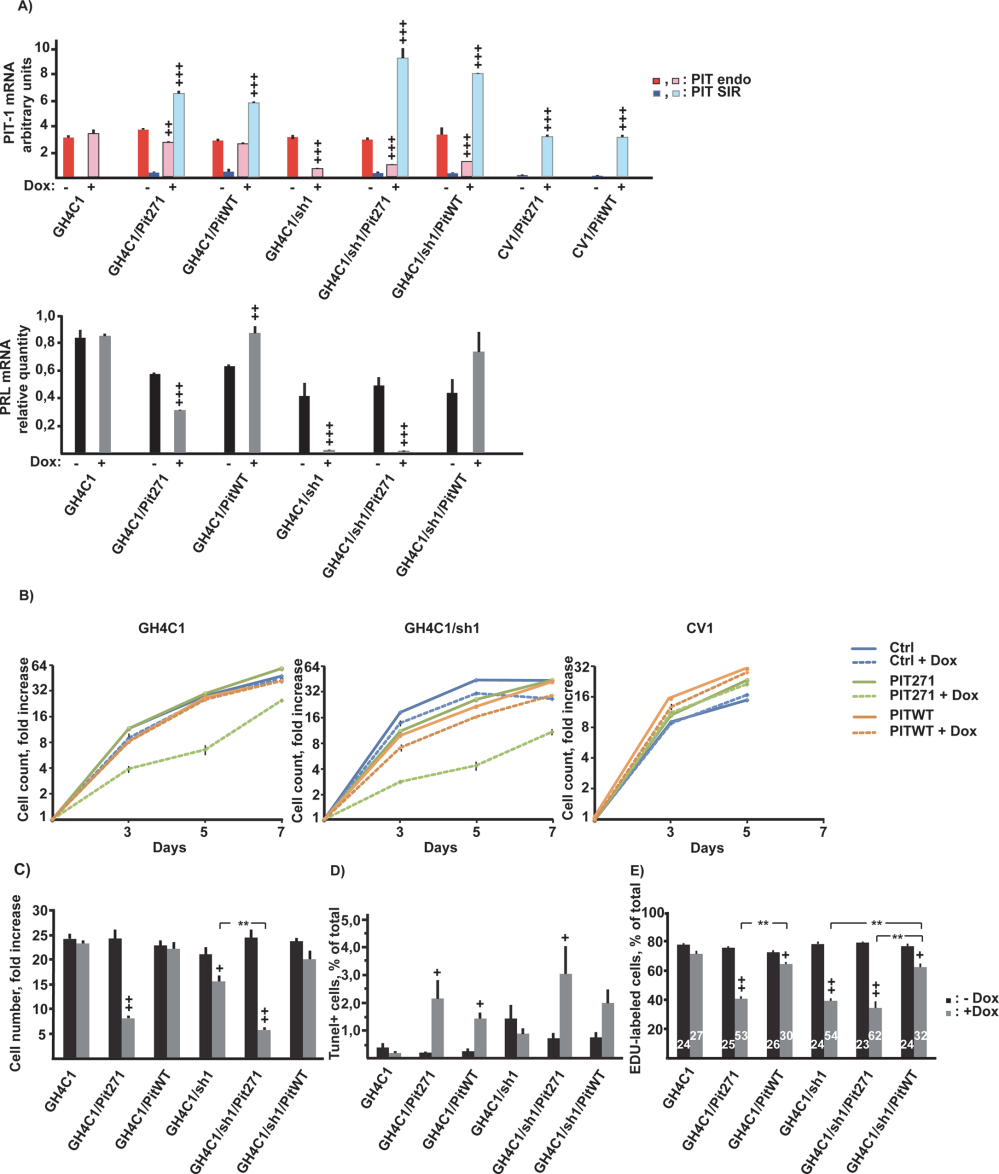
Effects of PITWT, PIT271 and/or shRNA for PIT-1 on the proliferation and survival of GH4C1 cells. A. Expression of mRNA's for PIT-1 or PIT271 (upper panel), as well as for PRL (lower panel), in the different cell lines in the absence or presence of 1 μg/ml doxycycline for 5 days. The cells used were from the same experiment as those grown on parallel plates to measure proliferation rate (Fig. 3B). Results for 'Pit endo' and ‘Pit SIR’ have been obtained by normalizing the values obtained in qPCR to those obtained with known quantities of external controls, and can thus be compared. Values correspond to mean ± SEM (n = 3). The significance of the effect of docycycline-induction on the expression of endogenous PIT-1, of the transgene or PRL was evaluated by the Student's t-test. +: p<0,05; ++: p<0,01; +++: p<0,001. B. Effect of the expression of PITWT, PIT271 and/or shRNA for PIT-1 on the proliferation of GH4C1 or GH4C1-sh1 cells as well as on CV1 cells. Cells were counted on triplicate wells. Induction of the expression of the shRNA (GH4C1-sh1 cells) and of the transcription factors was started on day 0 and continued throughout the experiment. 'Ctrl' represent GH4C1, GH4C1-sh1 or CV1 cells not infected with pInducer-20 vector expressing a transcription factor. Counting of CV1 cells was discontinued after 5 days, as cells became too confluent. Values correspond to mean ± SEM (n = 3). C. Effect of the expression of PITWT, PIT271 and/or shRNA for PIT-1 on the proliferation of GH4C1 or GH4C1-sh1 cells. Data represent the pooled results of the four experiments (with triplicate wells in each) of which one is illustrated on Fig. 3B, and correspond to the cell numbers found in the absence or the presence of doxycycline five day (D5) after the start of the induction. Mean ±SEM, +: p<0,05, ++: p<0,01, relative to the same population without doxycycline, *: p<0,05. D. Effect of the expression of PITWT, PIT271 and/or shRNA for PIT-1 on DNA fragmentation as evaluated by Tunel assay following 4 days exposure to doxycycline. Labeled and unlabeled cells were counted from 6 micrographs taken at random from duplicate wells and the experiment was repeated twice. Mean ±SEM, +: p<0,05, relative to the same population without doxycycline. E. Effect of the expression of PITWT, PIT271 and/or shRNA for PIT-1 on the division of GH4C1 or GH4C1-sh1 cells using EDU as cell division marker. EDU was present for 15–17h between day 3 and 4 of the induction by doxycycline. Labeled and unlabeled cells were counted from 6 micrographs taken at random from duplicate wells and experiments were repeated three times. Numbers given within the columns correspond to the population doubling time in hours as calculated from the labeling percentages. Mean ±SEM, +: p<0,05, ++: p<0,01, relative to the same population without doxycycline, **: p<0,01.

**Fig 4:**
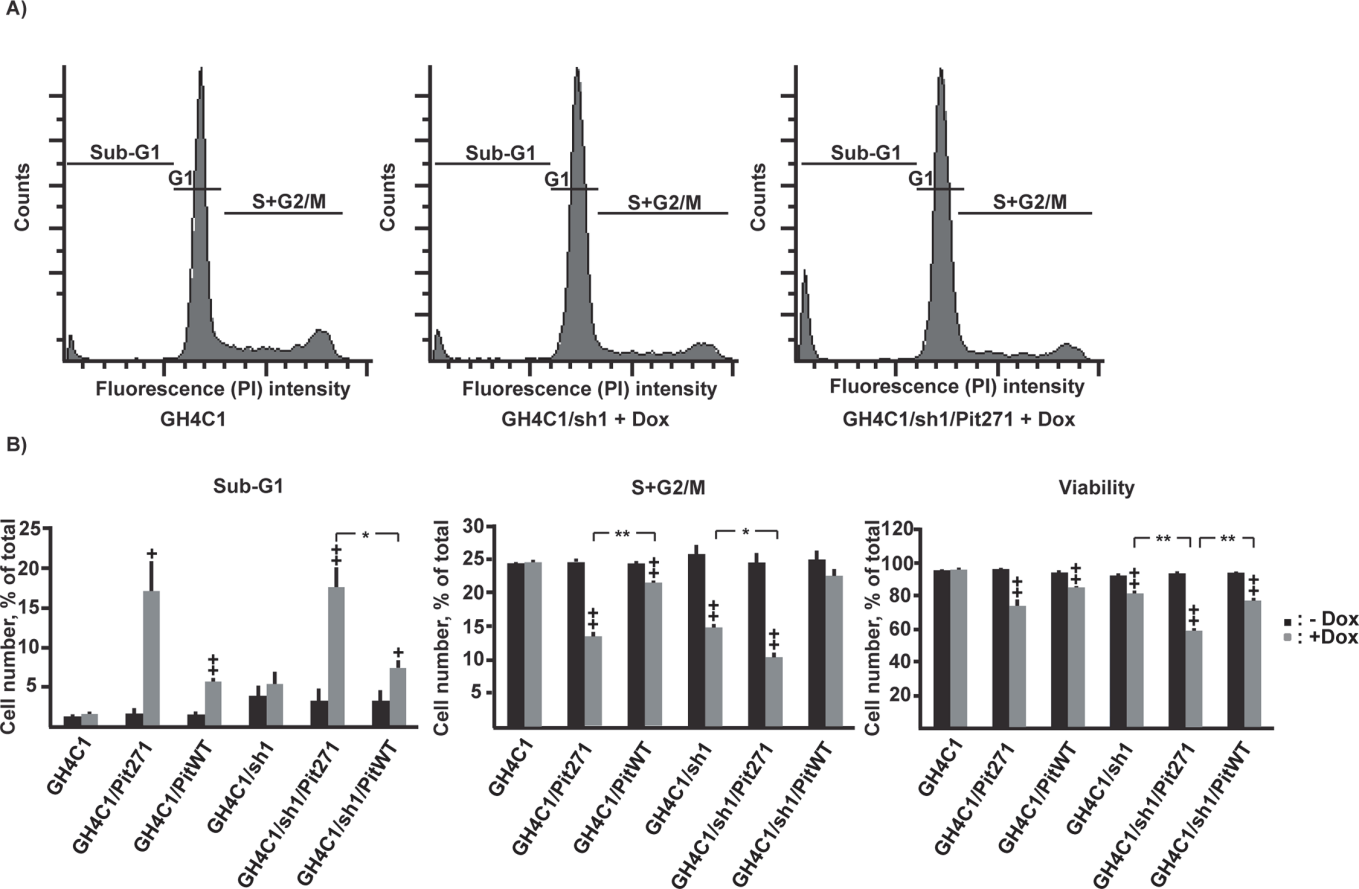
Flow cytometer analysis of the different cell populations in the absence or presence of 1 μg/ml doxycycline for 5 days. A. Representative examples of histograms based on DNA content showing the decrease of the proportion of the S+G2/M population upon induction of shRNA or PIT271, with the concurrent increase in the sub-G1 population in the latter case. B. Quantitative analysis of the different sub-populations. Results (mean ± SEM) are pooled from four independent experiments, with duplicate measures in each. +: p<0,05, ++: p<0.01, relative to the same population without doxycycline, *: p<0,05, **: p<0,01.

To evaluate global cell proliferation, the different cell lines obtained previously were cultured in the absence or the presence of the inducer doxycycline. The treatment elicited the expected changes in *Pit-1* and *Prl* gene expression (Fig. 3A), consistent with what we have observed previously when developing the cell lines (Fig. 2E and 2F). Note in particular the decrease of the expression of endogenous PIT-1 and of PRL following the induction of PIT271 in GH4C1 cells, consistent with its known dominant negative character. Doxycycline treatment has no effect on its own on the different parameters examined as shown by the results obtained for the parent GH4C1 cells (see Figs. 3 and 4).

Inhibition of the expression of endogenous *Pit-1* following induction of shRNA led to a slight, but significant, inhibition of cell growth on its own (Fig. 3B central panel and 3C). This effect was reversed by the expression of exogenous PITWT. Note that, while the results presented on Fig. 3B and 3C were obtained using sh1/A2, the same inhibition of cell growth was observed with GH4C1 cells harboring the unrelated sequence sh3 (clone sh3/A3; results not shown).

Expression of PIT271 in GH4C1 cells led to an inhibition of cell growth that was markedly stronger than that seen when the expression of endogenous PIT-1 was blocked by the shRNA (Fig. 3B and 3C). This effect of PIT271 was present also when its expression was induced concurrently with the induction of shRNA and the resulting inhibition of the expression of endogenous PIT-1 (Fig. 3B central panel), suggesting that the effect of PIT271 is independent from its possible interaction with, and blockade of, PIT-1. Finally the non-pituitary cell line CV1 was unaffected by the expression of either PITWT or PIT271 (Fig. 3B right panel).

The slower growth induced by the shRNA or by PIT271 could be related to an increase in cell death, a decrease in cell division rate or both. In our previous work we documented that PIT271 induced cell death in GH4C1 cells that was accompanied by DNA fragmentation, suggesting the existence of apoptosis [11]. Using Tunel assay, we examined whether it is also the case when endogenous PIT-1 expression is blocked or when PIT271 is expressed in the absence of endogenous PIT-1 (Fig. 3D). The blockade of PIT-1 expression by shRNA did not increase the proportion of Tunel-positive cells. On the contrary the expression of PIT271 led to a significant increase of Tunel labeling in GH4C1 cells, confirming our previous results. Similar to its effect on cell proliferation, the action of PIT271 on DNA fragmentation was independent on the expression of endogenous PIT-1, as it was observed also in cells in which endogenous PIT-1 expression was blocked by shRNA. Surprisingly, expression of exogenous PITWT, resulting in a higher than normal total PIT-1 expression in the cells (Fig. 3A) led also to a significant increase in Tunel labeling.

To examine cell division, we evaluated the incorporation of a cell division marker, EDU, in the different populations of cells and under different conditions (Fig. 3E). Cell division rate was markedly decreased by PIT271, both in the presence or absence of expression of endogenous PIT-1. The inhibition by shRNA of endogenous PIT-1 led in itself to a decrease of cell division rate, of comparable magnitude as that observed with PIT271, and this decrease was significantly reversed, albeit not totally, by the expression of exogenous PITWT. However, and somewhat paradoxically, the overexpression of PIT-1 in GH4C1 cells led also to a significant, though milder, depression of cell division.

To confirm these results by a different approach, we have performed, in a separate set of experiments, an analysis by flow cytometry. The results of this analysis (Fig. 4) confirmed those detailed above and reproduced closely the results shown on Fig. 3D, showing an increase of cell death (increase of the proportion of sub-G1 population) by PIT271 and, to a lesser extent, by PITWT overexpression. Conversely, cell division (proportion of the S+G2/M population) was decreased in the same conditions, but also by the blockade of endogenous PIT-1 expression, reproducing the results we have obtained using EDU incorporation (Fig. 3E). Finally, viability of the cells was found to be decreased by the expression of PIT271 or PITWT, but also by the blockade of the expression of endogenous PIT-1.

### Dose-dependence of the effects of shRNA, PITWT and PIT271

**Fig 5:**
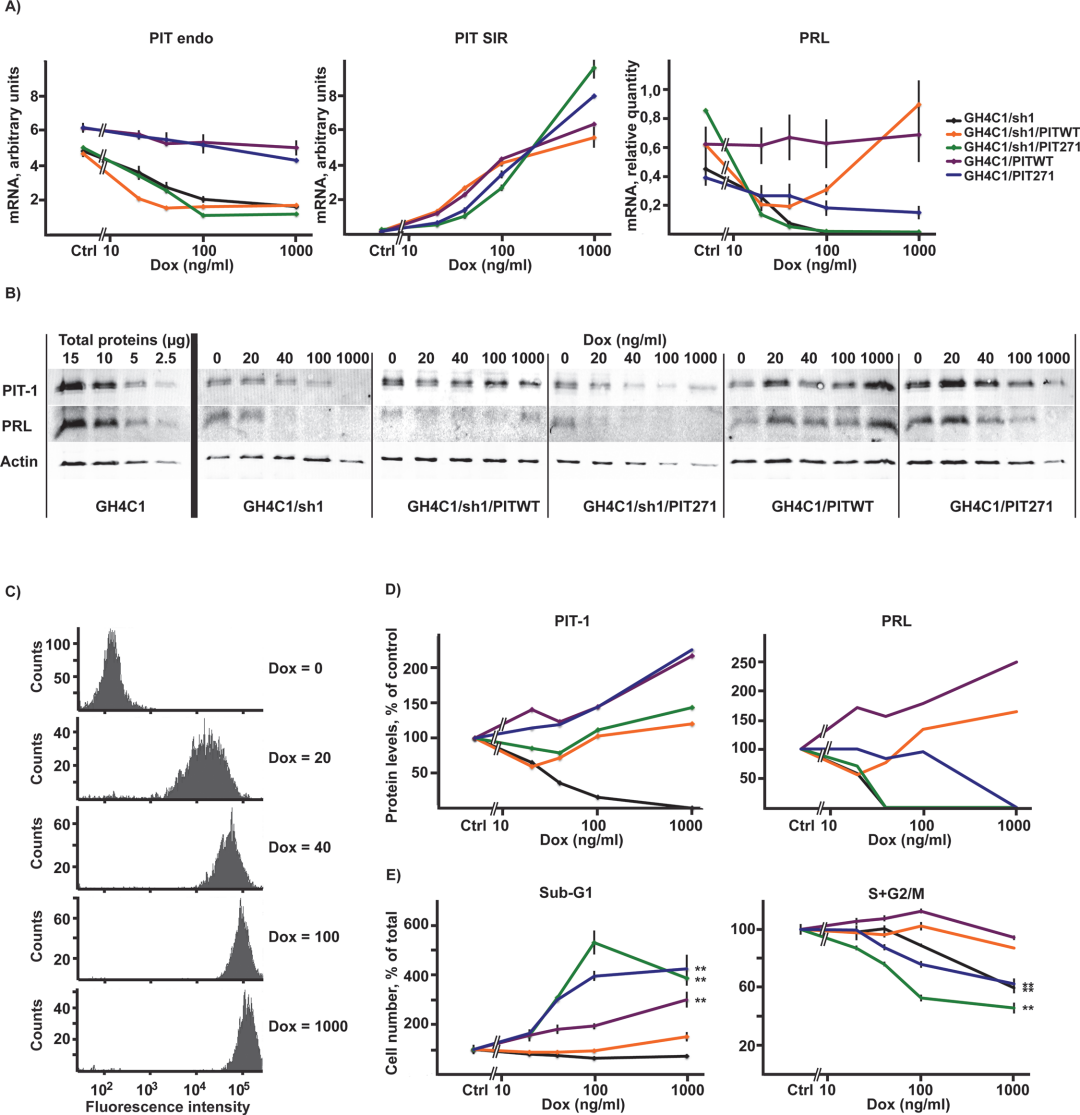
Dose-dependence of the actions of shRNA, PITWT and PIT271. A. Dose-responses curves of mRNA's for PIT-1 and PRL, in the different cell lines grown in presence of different doses (0, 20, 40, 100 or 1000 ng/ml) of doxycycline for 5 days. Results for 'PIT endo' and ‘PIT SIR’ have been obtained by normalizing the values obtained in qPCR to those obtained with known quantities of external controls, and can thus be compared. The values at 'Ctrl' correspond to the levels measured in cells exposed to 0 ng/ml doxycycline. The results correspond to the mean ± SEM of three independent measurements. B. Western blots for cells included in the experiment depicted in Fig. 5A. C. Representative examples of histograms based on the fluorescence of RFP as evaluated by flow cytometry in GHC1/PITWT cells grown in presence of different doses of doxycycline for 5 days. D. Quantitation of Western blots of Fig. 5B. Bands of the protein of interest were normalized relative to actin and expressed as percentage of control cells grown in the absence of doxycycline. Legends are the same as for Fig. 5A. E. Dose-response curves for the proportions of the sub-G1 and the S+G2M populations in the different cell lines grown in presence of different doses (0, 20, 40, 100 or 1000 ng/ml) of doxycycline for 5 days. The values at 'Ctrl' correspond to the levels measured in cells exposed to 0 ng/ml doxycycline. The results are given as mean ± SEM (n = 3), significant correlations (**: p<0.001) are indicated beside the corresponding curves. Legends are the same as for Fig. 5A. Pearson coefficient of correlations were for Sub-G1 results: GH4C1/PIT271: r = 0,9449 (df = 10), GH4C1/PITWT: r = 0,8144 (df = 13), GHC1/sh1/PIT271: r = 0,9668 (df = 13) and for M+G2M results: GHC1/PIT271: r = -0,8044 (df = 13), GH4C1/sh1: r = -0,9615 (df = 13), GH4C1/sh1/PIT271: r = -0,9802 (df = 10).

We examined whether the effects of shRNA, PITWT and of PIT271 are dependent on the level of expression of these factors. For that, we first checked whether the level of their expression could be modulated by the concentration of doxycycline during induction. We observed indeed that the degree of induction was strictly dose-dependent between 0 and 1000 ng/ml doxycycline (Fig. 5A). The increasing level of transcription of the transgenes was accompanied by parallel changes in the levels of PIT-1 protein and in the expression its target PRL (Fig. 5B and 5D). Moreover, we observed, using GH4C1/sh1 cells and flow-cytometry, that the expression of RFP (the transcription of which is under the control of the same promoter as that for the above transgenes, see Fig. 1A) presented a unimodal distribution at all doxycycline concentrations, with an increase of the mean level of expression with increasing doxycycline concentrations (Fig. 5C). This indicates that the increase in the transcription of the transgenes with increasing levels of the inducer, as observed by the global analyses done by RT-qPCR or Western-blots, was related to a graded effect in the individual cells of the population, rather than to an all-or-none effect affecting a small subpopulation that would be more sensitive to the inducer and the proportion of which would increase with increasing doses of doxyxycline.

The magnitudes of the death-inducing effect of PITWT or PIT271 as well as of the decrease of division rate induced by PIT271 and the blockade of expression of endogenous PIT-1 by shRNA were all dependent on the level of expression of the transgenes as induced by increasing doses of doxycycline, and increased as the level of induction increased. The correlation with the dose of doxycycline was statistically significant for each of these effects. Moreover, the effects were observable even at the lowest dose (20 ng/ml) of the inducer (Fig. 5E), and we could not observe a threshold level of induction under which there would be no effect on cell death and/or division. Finally, the results show that PIT271 is considerably more potent in inducing death of the cells than PITWT. Taking the example of cells grown in the presence of 100 ng/ml doxycycline, cell death was found to be twice as important when PIT271 was induced (compare the proportion of the Sub-G2 population in GH4C1/PITWT and GH4C1/PIT271 cells, Fig. 5E), although the level of PIT271 can be estimated to be only half of that of PITWT (Fig. 5A and 5D).

### Mechanisms of cell death induced by PITWT or PIT271

**Fig 6:**
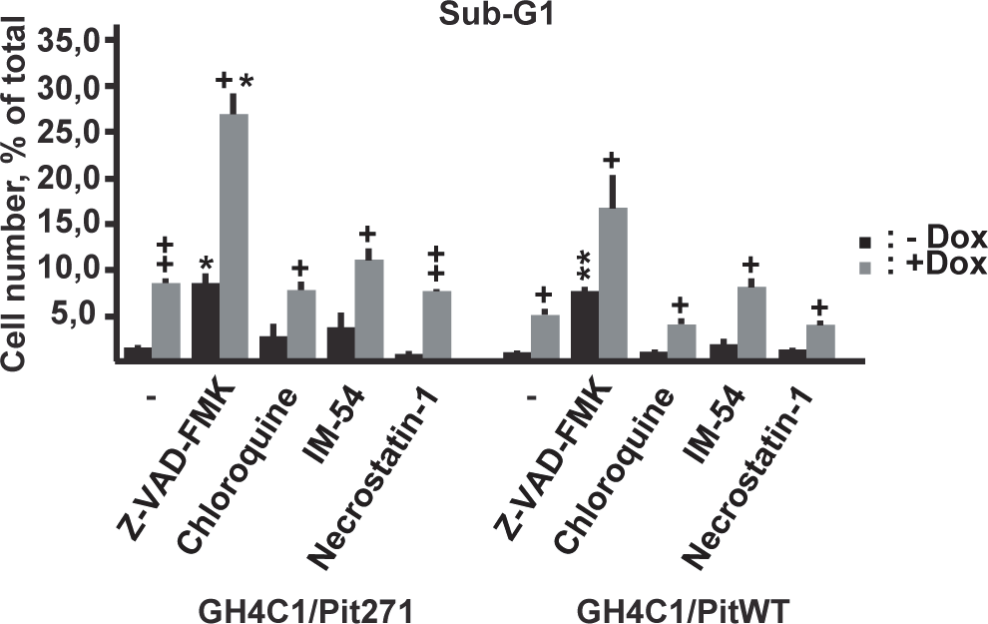
Effect of antagonists of different types of cell death on the PITWT or PIT271 induced cell death. The proportion of sub-G1 population was evaluated by flow cytometry. Drugs were Z-VAD-FMK (25 μM), a general caspase inhibitor; chloroquine (10 μM), an inhibitor of autophagy; IM-54 (10 μM) and necrostatin-1 (10 μM), inhibitors of necrosis. Flow cytometry was performed 4 days after the addition of the drugs together with 1 μg/ml doxycycline. Results (mean ±SEM) are pooled from two independent experiments, with triplicate measures in each. +: p < 0,05, ++: p < 0,01 relative to the same population without doxycycline; *: p < 0,05, **: p < 0,01 relative to the same condition in the absence of drug.

To get some insight into the mechanisms of the cell death induced by the overexpression of PITWT and by PIT271 that we have observed in our previous experiments, we tried to block these effect by pharmacologic inhibitors considered to target specific kinds of cells death: Z-VAD-FMK, a general caspase inhibitor blocking caspase-dependent apoptosis, chloroquine, targeting autophagy, and two drugs blocking necrosis, IM-54 and necrostatin-1. Drugs were present throughout the induction of PITWT or PIT271. The effects of the drugs were tested by examining the increase of the sub-G1 population following induction of PIT271 or PITWT in GH4C1 cells, without expression of shRNA, to avoid a possible interference from the blockade of endogenous PIT-1 expression. However, none of the drugs tested modified significantly the induced cell death (Fig. 6.). It can be noted that Z-VAD-FMK itself induced cell death in control cells, as judged by the increase of the proportion of the sub-G1 population, similar to what has been observed for some other cell types [18–20]. However, PIT271 or PITWT induced a marked cell death that was clearly superimposed on that induced by the drug alone.

In a previous study we have identified, using ChIP-Chip, a number of putative direct targets of PIT-1 [21]. We examined, using RT-qPCR, whether genes known to be involved in various cell death mechanisms and/or cell division and identified in these experiments (*Aatf*, *Bag1*, *Bag3*, *Ccni*, *Dad1*, *Dap*, *Nudc*, *Rad17*, *Serinc3*, *Sod2*, *Tnfrsf1a and Vcp*) were activated during the induction of cell death by PIT271 or PITWT. However, we could not detect any significant change in the expression of any of these factors following the induction of either PITWT or PIT271. Neither could we detect any activation of P19ARF or P53, described earlier to be involved in the cell death induced by a RET-PIT-1 pathway [12], be it by RT-qPCR or by Western blots, using the same antibodies as those authors as well as another antibody.

### Decrease of viability of human tumor cells by PITWT

**Fig 7:**
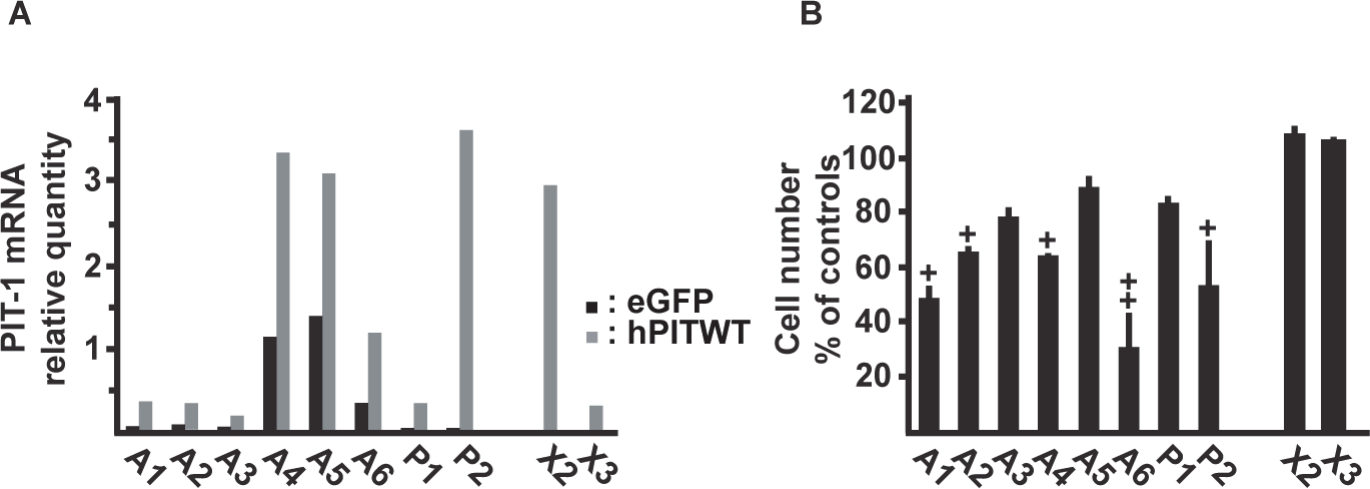
Effects of hPITWT on the viability of primary human pituitary tumor cells. A. Expression of mRNA's for PIT-1 in individual tumor populations following infection with the control, eGFP-expressing lentiviral vector or with the hPITWT expressing lentiviral vector. The global increase in the expression of PIT-1 mRNA following the infection was statistically significant (paired t-test, p<0,005). B. Viability of individual tumor populations following infection with the hPITWT expressing lentiviral vector, expressed relative to the viability following infection with the control, eGFP-expressing lentiviral vector, as evaluated by the Cell Titer-Glo assay. Mean ± SEM (n = 3), +: p < 0,05, ++: p < 0,01.

We have recently shown that PIT271 induces, similar to what we have observed previously in the GH4C1 cell line [11], death of primary human tumor cells [10]. We wished to test whether the adverse effect of PITWT on cell viability described here could also be reproduced in such tumor cells. Cells were infected with an hPITWT-expressing lentiviral vector and the influence on viability and cell death was explored 9 days post-infection. Note that, considering the very large variations of the different parameters among different tumors, results are given individually for each tumor. The infection led to a clearcut, though variable among tumors, increase in the quantities of PIT-1 mRNA (Fig. 7A) that was highly significant (t = 5,39, ddf = 9, p = 0,004, using Student's t-test for paired data). Note that in these experiments we could not separate transcription of *Pit-1* from the lentiviral vector from the transcription of endogenous *Pit-1*. While the reaction of the different adenomas was variable, altogether there was a significant decrease of viability of the somatolactotroph/lactotroph adenomas following infection with the hPITWT expressing lentiviral vector as compared to cells infected with an eGFP expressing vector (Fig. 7B). The magnitude of this effect was comparable to that observed previously following the transduction of hPIT271 [10] and does not seem to correlate with the level of endogenous hPITWT expression (in the absence of infection) of the individual tumors (Fig. 7A). Viability of non-functional adenomas was not significantly affected by the expression of PITWT.

## Discussion

The present study reconciles the two, seemingly divergent, sets of results reported in the literature, and shows that PIT-1 is indeed required for the maintenance of somatolactotroph cells, as suggested by the initial observations of Castrillo *et al*. [9], but that it can also, when overexpressed, decrease cell proliferation and induce cell death, confirming thus the results reported by the group of C.V. Alvarez [12,13]. The decrease of cell viability induced by PIT-1 could be observed not only in the rat GH4C1 somatolactotroph cell line, but also in primary human somatolactotroph tumor cells. A further finding of our study is that while PIT271 induces both a decrease of cell division and death of the cells, confirming our earlier results [11], these effects, at variance with earlier interpretations, including ours, are independent of the presence of PIT-1 in the cells, i.e. are not the result of a blockade of PIT-1's action by PIT271.

This study was made possible by the development of a new cellular model based on the utilization of inducible lentiviral vectors, and permitting the silencing of endogenous PIT-1 through the expression of a specific shRNA on the one hand, and the expression of exogenous normal or mutant PIT-1 on the other hand. In particular, by combining the two kinds of vectors, we were able to exchange the expression of endogenous PIT-1 for an exogenous factor. More generally, our results demonstrate that this approach may be used to exchange, in an inducible, dose-dependent and reversible manner, an endogenous transcription factor (or, as a matter of fact, any protein) for a mutant variant in cultured cells, allowing the study of the latter without interference by the endogenous wild type factor.

The molecular data obtained for the characterization of this model are in agreement with the known effects of PITWT and PIT271 and validate the model. Thus, blockade of the expression of endogenous PIT-1 leads to a dose-dependent decrease, and eventually collapse, of the expression of its target PRL. Further, expression of PIT271 in GH4C1 cells leads to the dose-dependent decrease of the expression of both PIT-1 and of PRL, confirming its dominant negative character [4,11,22]. Note that following the expression of shRNA or PIT271 in the cells the expression of PRL fell more precipitously than that of PIT-1 (see Fig. 5).

Our results show that an approximately twofold increase of PIT-1 expression (see Fig. 3A) induces mortality of somatolactotroph cells. This induction of cell death by PIT-1 seems to be less important in our hand than that reported by C.V. Alvarez [12,13]. However, the comparison is not straightforward, as in those publications the quantitation of cell death was based on counting the proportion of cells with condensed and fragmented chromatin, while we used flow cytometry or Tunel reaction. More importantly, the level of PIT-1 reached in their experimental setup is likely to be significantly higher (they document a tenfold increase in *Pit-1* mRNA, while with our conditions this increase, as well as that of the level of the protein itself, is around twofold), but only transient, peaking at 24h post-transfection and returning to control levels 48h post-transfection, when cell death was evaluated, while in our hand there is an elevated level of *Pit-1* mRNA lasting the whole duration of the experiment, in the present case 5–7 days. Nevertheless, our two sets of studies are in agreement as to the general, qualitative aspect of the effect of PIT-1.

The mechanisms of the cell death induced by PIT-1 overexpression, and the same can be said concerning of the cell death induced by PIT271 (see also below), are at this point not clear. Indeed, in the present experiments we have been unable to identify the direct target(s) on which PIT-1 (or PIT271) acts and that set in motion the process leading to cell death. Thus, we could not observe a modification of the level of expression of a number of potential direct targets of PIT-1 identified in our earlier work [21]. We have also been unable to confirm the increase of P19ARF expression reported earlier [12,13], be it at the level of its mRNA or the protein. Differences in the experimental conditions (in particular 24 to 48 h deprivation of serum in the experiments of Alvarez *et al*. [12,13], presence of normal concentrations of serum in ours) might explain this discrepancy. Further studies will be needed to identify the immediate targets of PIT-1 or PIT271 that are responsible for the initiation of the death process in the present conditions. Besides the primary targets, the question of the death mechanisms involved remains also open. Concerning the type of cell death elicited by PIT-1 or PIT271, our results suggest that it is not related to an autophagic or necrotic process, and the increase of Tunel labeling suggests an apoptotic, although caspase-independent, mechanism, as shown earlier for the cell death induced by PIT271 [11]. This mechanism seems to be at play not only in GH4C1 cells, but also in human somatolactotroph adenomas, as in a preliminary experiment using two such adenomas, we observed that hPITWT expression increases Tunel-labeling in these cells (results not shown). Given the simultaneous increase of cell death and decrease in division rate induced by PIT-1 or by PIT271, we expected to confirm the involvement of P53 in this process, and the lack of change in P53 expression in the present experiments following induction of PITWT or PIT271 is somewhat surprising. Indeed, P53 is an important hub in the regulatory network mediating the increase of cell death and concurrent decrease of cell division observed in situations of genotoxic stress or disruption of certain signaling pathways [23,24]. However, activation of p53 involves also various post-transcriptional modifications as well as interaction with cofactors, and future studies will have to investigate whether PIT-1 overexpression or PIT271 activate the P53 pathway through such mechanisms.

Maintenance of division of GH4C1 cells requires a normal expression of PIT-1 as shown by the marked fall in proliferation rate when expression of endogenous PIT-1 is blocked by shRNA. This action of shRNA is not accompanied by cell death, indicating that the mechanisms involved in this effect are different from those at play in the proliferation-depressing action of elevated PIT-1 that include also cell death. Basal PIT-1 level might directly regulate, in normal cells, expression of some yet to be identified gene(s) involved in cell division to maintain normal proliferation rate. On the other hand, PIT-1 is known to interact with a large number of transcription factors and cofactors, some of which are involved in the regulation of the proliferation of pituitary cells [25–29] and interactions with some of these factors, or other yet to be identified factors may be involved in the proliferation-maintaining action of PIT-1.

Taken globally, our result suggest that there is an optimal level of PIT-1 expression in GH4C1 cells and that any deviation from this set point, even if relatively moderate, have detrimental consequences for cells, a downward shift leading to a marked decrease of cell division rate, and an upward deviation to cell death accompanied by some decrease of cell division. Thus, besides its well-characterized role in regulating its hormonal targets, PIT-1 seems to be involved in the maintenance of proliferative activity on the one hand, and in the death of the cells expressing it at high level on the other hand. This differential action of PIT-1 may be determined by a concentration-dependent binding to recognition sequences of different affinities and/or by a concentration-dependent interactions with different cofactors, as documented for other transcription factors [30–34].

Physiology may offer a clue to understand the biological significance of this Janus-faced function of PIT-1. Indeed, there is, in the developing pituitary, an early postnatal wave of division of somatolactotroph cells [35,36], resulting in the marked expansion of this population. During late pregnancy and lactation, the lactotroph population expands secondarily though cell division [37], while at weaning there is an involution of this population, in part through cell death [38–41]. It can be hypothesized that PIT-1 participates, through its dual function, in both of these processes. Thus it could contribute to maintain the proliferation of the somatolactotroph population during development or lactation. On the other hand, an elevated PIT-1 expression, possibly induced by RET [12,13,38], might be instrumental in triggering the post-weaning decrease of lactotrophs through cell death. Note, however, that cell death seems to be more sensitive to the levels of PIT-1, being elevated already for a slight (around 20%) increase in intracellular PIT-1 level, while for cell division to decrease noticeably the expression of PIT-1 must diminish by 60–80% (Fig. 5.). This observation suggests that the primary effect of PIT-1, besides the regulation of the expression of its classical hormonal targets, such as *Prl*, *Gh* or *Tsh*, might be the regulation of cell death.

For PIT271, our results show that its cell-death inducing effect is unrelated to an antagonization of the action of endogenous PIT-1. Indeed, on the one hand cell death is induced even in the absence of expression of endogenous PIT-1, and on the other hand the blockade of PIT-1 expression by shRNA by itself does not induces death of the cells. One could argue that the effect of PIT271 in the GH4C1/sh1/PIT271 cells is related to its antagonization of the residual PIT-1 that exists in the cells even following the maximal induction of the shRNA. However, this residual expression of PIT-1 seems to be too low to have a functional effect, as judged by the level expression of PRL (Fig. 5). Moreover, cell death induced by PIT271 at low doses of doxycycline is of the same magnitude in the GH4C1/PIT271 and the GH4C1/sh1/PIT271 cells, even though the ratio of PIT271 to endogenous PIT-1 protein in the cells is very different in these two cases, as shown by the inspection of Fig. 5. This support our hypothesis that the death-inducing effect of this mutant is an action of its own, independent of its dominant negative character, and similar to that of PIT-1. Conversely, at this point we cannot exclude that the strong decrease in cell division elicited by the expression of PIT271 is explained by an interaction/competition with endogenous PIT-1, as the same effect, of approximately the same magnitude, can be observed when the expression of endogenous PIT-1 is blocked by shRNA.

The question that arises then is whether the cell death induced by the expression of PIT271 or by the overexpression of PIT-1 are mediated by the same mechanisms. From a phenomenological point of view, the global characteristics of the cell death induced by these two variants of PIT-1 seem to be similar (see above). However, the magnitude of cell death is considerably larger when induced by PIT271, despite a similar level of expression of PIT-1 and PIT271 following the induction (see Figs. 3A, 5A, 5B, 5D). This observation suggests that either PIT271 influences the same targets as PIT-1 but, related maybe to the R271W mutation, has a greater efficacy, or that the two have different mechanisms of actions. An indirect argument in favor of a common mechanism is that the cell-death inducing effect of PITWT and PIT271 are both specific for somatolactotroph cells and cannot be observed in other cell types. However, this point will need to be clarified by further studies aiming at elucidating and comparing the molecular mechanisms of the action of PIT-1 and PIT271.

PIT271 is considered as a dominant negative mutant of PIT-1, as heterozygote patients, i.e. expressing one normal allele of PIT-1 besides the mutant allele, suffer from CPHD. Note that the GH4C1/PIT271 cells exposed to 40 ng/ml doxycycline mimic this situation, as in this condition, in which we have a threefold increase in cell death (see Fig. 5E) we can estimate to have about half the normal level of endogenous PIT-1 and an equivalent expression of PIT271 in the cells. The mechanism of this effect is not clear, however. It was hypothesized that it could be explained by PIT271 interfering with the formation of productive PIT-1 dimers on its target promoters [42] and/or by the defective recruitment of cofactors on the promoter as documented for a closely related mutant, PIT-1(R271A) [43]. According to these hypothesis, PIT271 could schematically considered as competing out endogenous PIT-1 and its pathological effect would be related to a overall loss of PIT-1’s efficacy due to this competition. Our present result demonstrate that PIT271 has major physiological effects—induction of cell death, possibly decrease of cell division- that are specific to this mutant, and independent of the presence of, and interactions with, PIT-1. These effects can very well explain the pathogenic influence of PIT271, without the need to invoke a competition with PIT-1, as they can lead on their own to the loss of the somatolactotroph cell population characterizing the patients carrying this mutation, and, as our results show, this effect would be present even in the presence of an expression of an equivalent level of PIT-1, as it is the case in heterozygote patients. On the other hand, it must be stressed that PIT271 does decrease the activation by PIT-1 of its target genes *Prl* and *Gh* (see Figs. 3A, 5A, 5B and 5D for its action on *Prl* or [11]) and thus have also a true dominant negative “competitive” action. This action could contribute to the observed clinical traits of CPHD in patients.

A final noteworthy aspect of our result is the cell-type specificity of the observed effects. Indeed the blockade of cell proliferation by PIT-1 or of PIT271 is specific for somatolactotroph cells, and we could observe no effect in CV1 or non-somatolactotroph human tumor cells (present study), or, for PIT271, in HeLa cells [11]. This suggests that this action is dependent on specific cell-type dependent conditions and may require the cooperation with a somatolactotroph cell-specific factor. Experiments are in progress in our laboratory to identify factors that might explain this specificity.

## References

[1] Ingraham HA, Chen RP, Mangalam HJ, Elsholtz HP, Flynn SE, Lin CR, et al A tissue-specific transcription factor containing a homeodomain specifies a pituitary phenotype. Cell. 1988; 55: 519–29. 2902928 10.1016/0092-8674(88)90038-4

[2] Bodner M, Castrillo JL, Theill LE, Deerinck T, Ellisman M, Karin M. The pituitary-specific transcription factor GHF-1 is a homeobox-containing protein. Cell. 1988; 55: 505–18. 2902927 10.1016/0092-8674(88)90037-2

[3] Andersen B, Rosenfeld MG. POU domain factors in the neuroendocrine system: Lessons from developmental biology provide insights into human disease. Endocr Rev. 2001; 22: 2–35. 11159814 10.1210/edrv.22.1.0421

[4] Radovick S, Nations M, Du Y, Berg LA, Weintraub BD, Wondisford FE. A mutation in the POU-homeodomain of Pit-1 responsible for combined pituitary hormone deficiency. Science. 1992; 257: 1115–8. 1509262 10.1126/science.257.5073.1115

[5] Pfäffle RW, DiMattia GE, Parks JS, Brown MR, Wit JM, Jansen M, et al Mutation of the POU-specific domain of Pit-1 and hypopituitarism without pituitary hypoplasia. Science. 1992; 257: 1118–21. 1509263 10.1126/science.257.5073.1118

[6] Romero CJ, Nesi-França S, Radovick S. The molecular basis of hypopituitarism. Trends in Endocrinology Metabolism. 2009; 20: 506–16. 10.1016/j.tem.2009.06.005 19854060 PMC2787976

[7] Li S, Crenshaw EB, Rawson EJ, Simmons DM, Swanson LW, Rosenfeld MG. Dwarf locus mutants lacking three pituitary cell types result from mutations in the POU-domain gene Pit-1. Nature. 1990; 347: 528–33. 1977085 10.1038/347528a0

[8] Ward RD, Stone BM, Raetzman LT, Camper SA. Cell proliferation and vascularization in mouse models of pituitary hormone deficiency. Mol Endocrinol. 2006; 20: 1378–90. 16556738 10.1210/me.2005-0409

[9] Castrillo JL, Theill LE, Karin M. Function of the homeodomain protein GHF1 in pituitary cell proliferation. Science. 1991; 253: 197–9. 1677216 10.1126/science.1677216

[10] Roche C, Rasolonjanahary R, Thirion S, Goddard I, Fusco A, Figarella-Branger D, et al Inactivation of transcription factor Pit-1 to target tumoral somatolactotroph cells. Hum Gene Ther. 2012; 23: 104–14. 10.1089/hum.2011.105 21942649

[11] Pellegrini I, Roche C, Quentien M-H, Ferrand M, Gunz G, Thirion S, et al Involvement of the pituitary-specific transcription factor Pit-1 in somatolactotrope cell growth and death: An approach using dominant-negative Pit-1 mutants. Mol Endocrinol. 2006; 20: 3212–27. 16901973 10.1210/me.2006-0122

[12] Diaz-Rodriguez E, García-Lavandeira M, Perez-Romero S, Senra A, Cañibano C, Palmero I, et al Direct promoter induction of p19Arf by Pit-1 explains the dependence receptor RET/Pit-1/p53-induced apoptosis in the pituitary somatotroph cells. Oncogene. 2012; 31: 2824–35. 10.1038/onc.2011.458 22020338

[13] Cañibano C, Rodriguez NL, Saez C, Tovar S, Garcia-Lavandeira M, Borrello MG, et al The dependence receptor Ret induces apoptosis in somatotrophs through a Pit-1/p53 pathway, preventing tumor growth. EMBO J. 2007; 26: 2015–28. 17380130 10.1038/sj.emboj.7601636 PMC1852774

[14] Chang K, Elledge SJ, Hannon GJ. Lessons from nature: MicroRNA-based shRNA libraries. Nat Methods. 2006; 3: 707–14. 16929316 10.1038/nmeth923

[15] Meerbrey KL, Hu G, Kessler JD, Roarty K, Li MZ, Fang JE, et al The pInducer lentiviral toolkit for inducible RNA interference in vitro and in vivo. Proc Natl Acad Sci U S A. 2011; 108: 3665–70. 10.1073/pnas.1019736108 21307310 PMC3048138

[16] Roche C, Zamora AJ, Taieb D, Lavaque E, Rasolonjanahary R, Dufour H, et al Lentiviral vectors efficiently transduce human gonadotroph and somatotroph adenomas in vitro. Targeted expression of transgene by pituitary hormone promoters. J Endocrinol. 2004; 183: 217–33. 15525589 10.1677/joe.1.05759

[17] Gossen M, Bujard H. Tight control of gene expression in mammalian cells by tetracycline-responsive promoters. Proc Natl Acad Sci U S A. 1992; 89: 5547–51. 1319065 10.1073/pnas.89.12.5547 PMC49329

[18] Martinet W, Schrijvers DM, Herman AG, De Meyer GRY. Z-VAD-Fmk-induced non-apoptotic cell death of macrophages: Possibilities and limitations for atherosclerotic plaque stabilization. Autophagy. 2006; 2: 312–4. 16874073 10.4161/auto.2966

[19] Wu Y-T, Tan H-L, Huang Q, Sun X-J, Zhu X, Shen H-M. ZVAD-induced necroptosis in L929 cells depends on autocrine production of TNFα mediated by the PKC-MAPKs-AP-1 pathway. Cell Death Differ. 2011; 18: 26–37. 10.1038/cdd.2010.72 20539307 PMC3131876

[20] Chen S-Y, Chiu L-Y, Ma M-C, Wang J-S, Chien C-L, Lin W-W. ZVAD-induced autophagic cell death requires c-Src-dependent ERK and JNK activation and reactive oxygen species generation. Autophagy. 2011; 7: 217–28. 21127402 10.4161/auto.7.2.14212 PMC3039770

[21] Herman J-P, Jullien N, Guillen S, Enjalbert A, Pellegrini I, Franc J-L. Research resource: A genome-wide study identifies potential new target genes for POU1F1. Mol Endocrinol. 2012; 26: 1455–63. 10.1210/me.2011-1308 22638072 PMC5416979

[22] Cohen LE, Wondisford FE, Salvatoni A, Maghnie M, Brucker-Davis F, Weintraub BD, et al A "hot spot" in the Pit-1 gene responsible for combined pituitary hormone deficiency: Clinical and molecular correlates. J Clin Endocrinol Metab. 1995; 80: 679–84. 7852536 10.1210/jcem.80.2.7852536

[23] Vogelstein B, Lane D, Levine AJ. Surfing the p53 network. Nature. 2000; 408: 307–10. 11099028 10.1038/35042675

[24] Vousden KH, Prives C. Blinded by the light: The growing complexity of p53. Cell. 2009; 137: 413–31. 10.1016/j.cell.2009.04.037 19410540

[25] Baek SH, Kioussi C, Briata P, Wang D, Nguyen HD, Ohgi KA, et al Regulated subset of G1 growth-control genes in response to derepression by the Wnt pathway. Proc Natl Acad Sci U S A. 2003; 100: 3245–50. 12629224 10.1073/pnas.0330217100 PMC152277

[26] Voss JW, Wilson L, Rosenfeld MG. POU-domain proteins Pit-1 and Oct-1 interact to form a heteromeric complex and can cooperate to induce expression of the prolactin promoter. Genes Dev. 1991; 5: 1309–20. 2065979 10.1101/gad.5.7.1309

[27] Bach I, Rhodes SJ, Pearse RV, Heinzel T, Gloss B, Scully KM, et al P-Lim, a LIM homeodomain factor, is expressed during pituitary organ and cell commitment and synergizes with Pit-1. Proc Natl Acad Sci U S A. 1995; 92: 2720–4. 7708713 10.1073/pnas.92.7.2720 PMC42290

[28] Tremblay JJ, Lanctôt C, Drouin J. The pan-pituitary activator of transcription, Ptx1 (pituitary homeobox 1), acts in synergy with SF-1 and Pit1 and is an upstream regulator of the Lim-homeodomain gene Lim3/Lhx3. Mol Endocrinol. 1998; 12: 428–41. 9514159 10.1210/mend.12.3.0073

[29] Quentien MH, Manfroid I, Moncet D, Gunz G, Muller M, Grino M, et al Pitx factors are involved in basal and hormone-regulated activity of the human prolactin promoter. J Biol Chem. 2002; 277: 44408–16. 12223489 10.1074/jbc.M207824200

[30] Carotta S, Dakic A, D'Amico A, Pang SHM, Greig KT, Nutt SL, et al The transcription factor PU.1 controls dendritic cell development and Flt3 cytokine receptor expression in a dose-dependent manner. Immunity. 2010; 32: 628–41. 10.1016/j.immuni.2010.05.005 20510871

[31] Steger G, Corbach S. Dose-dependent regulation of the early promoter of human papillomavirus type 18 by the viral E2 protein. J Virol. 1997; 71: 50–8. 8985322 10.1128/jvi.71.1.50-58.1997 PMC191023

[32] Inga A, Storici F, Darden TA, Resnick MA. Differential transactivation by the p53 transcription factor is highly dependent on p53 level and promoter target sequence. Mol Cell Biol. 2002; 22: 8612–25. 12446780 10.1128/MCB.22.24.8612-8625.2002 PMC139870

[33] Takeuchi JK, Mileikovskaia M, Koshiba-Takeuchi K, Heidt AB, Mori AD, Arruda EP, et al Tbx20 dose-dependently regulates transcription factor networks required for mouse heart and motoneuron development. Development. 2005; 132: 2463–74. 15843409 10.1242/dev.01827

[34] Taranova OV, Magness ST, Fagan BM, Wu Y, Surzenko N, Hutton SR, et al SOX2 is a dose-dependent regulator of retinal neural progenitor competence. Genes Dev. 2006; 20: 1187–202. 16651659 10.1101/gad.1407906 PMC1472477

[35] Carbajo-Perez E, Watanabe YG. Cellular proliferation in the anterior pituitary of the rat during the postnatal period. Cell Tissue Res. 1990; 261: 333–8. 2401005 10.1007/BF00318674

[36] Taniguchi Y, Yasutaka S, Kominami R, Shinohara H. Proliferation and differentiation of pituitary somatotrophs and mammotrophs during late fetal and postnatal periods. Anat Embryol (Berl). 2001; 204: 469–75. 11876532 10.1007/s429-001-8003-x

[37] Yin P, Arita J. Differential regulation of prolactin release and lactotrope proliferation during pregnancy, lactation and the estrous cycle. Neuroendocrinology. 2000; 72: 72–9. 10971142 10.1159/000054574

[38] Guillou A, Romanò N, Bonnefont X, Le Tissier P, Mollard P, Martin AO. Modulation of the tyrosine kinase receptor ret/glial cell-derived neurotrophic factor (GDNF) signaling: A new player in reproduction induced anterior pituitary plasticity? Endocrinology. 2011; 152: 515–25. 10.1210/en.2010-0673 21239429

[39] Castrique E, Fernandez-Fuente M, Le Tissier P, Herman A, Levy A. Use of a prolactin-Cre/ROSA-YFP transgenic mouse provides no evidence for lactotroph transdifferentiation after weaning, or increase in lactotroph/somatotroph proportion in lactation. J Endocrinol. 2010; 205: 49–60. 10.1677/JOE-09-0414 20139144 PMC2837375

[40] Aoki A, de Gaisán EO, Pasolli HA, Torres AI. Disposal of cell debris from surplus lactotrophs of pituitary gland. Exp Clin Endocrinol Diabetes. 1996; 104: 256–62. 8817244 10.1055/s-0029-1211451

[41] Haggi ES, Torres AI, Maldonado CA, Aoki A. Regression of redundant lactotrophs in rat pituitary gland after cessation of lactation. J Endocrinol. 1986; 111: 367–73. 3805965 10.1677/joe.0.1110367

[42] Jacobson EM, Li P, Leon-del-Rio A, Rosenfeld MG, Aggarwal AK. Structure of Pit-1 POU domain bound to DNA as a dimer: Unexpected arrangement and flexibility. Genes Dev. 1997; 11: 198–212. 9009203 10.1101/gad.11.2.198

[43] Enwright JF, Kawecki-Crook Margaret A, Voss Ty C, Schaufele, Fred, Day, Richard N. A PIT-1 homeodomain mutant blocks the intranuclear recruitment of the CCAAT/enhancer binding protein alpha required for prolactin gene transcription. Mol Endocrinol. 2003; 17: 209–22. 12554749 10.1210/me.2001-0222 PMC2900764

